# Assessment tools for patients with stroke-related sarcopenia: a scoping review

**DOI:** 10.3389/fmed.2026.1709203

**Published:** 2026-08-31

**Authors:** Yanran Liang, Linli Xie, Wanwan Qiao, Runfang Guo, Hejia Wan, Jie Jing

**Affiliations:** 1 School of Medicine, University of Electronic Science and Technology of China, Chengdu, China; 2 Department of Operation Management, West China Hospital, Sichuan University, Chengdu, China; 3 Department of Geriatric Infection, Sichuan Provincial People’s Hospital, School of Medicine, Chengdu, China; 4 School of Nursing, Chengdu University of Traditional Chinese Medicine, Chengdu, China; 5 School of Nursing (Nursing School of Smart Healthcare Industry), Henan University of Chinese Medicine, Zhengzhou, China; 6 Department of Nursing, Sichuan Provincial People’s Hospital, School of Medicine, Chengdu, China

**Keywords:** functional outcome measures, geriatric neurology, muscle mass evaluation, rehabilitation medicine, sarcopenia assessment tools, scoping review, stroke-related sarcopenia

## Abstract

**Background:**

The assessment of stroke-related sarcopenia (SRS) is not easy due to the paralysis and functional limitations in mobility after stroke. There is a need to identify tools for the assessment of SRS that stroke patients can complete. Objectives: This review aimed to summarize the tools that can be used to assess SRS.

**Methods:**

Databases were systematically searched from the time of construction to 28 June 2026 based on the Arkensey and O’Malley framework. The databases searched included PubMed, Cumulative Index to Nursing and Allied Health Literature, Web of Science, Embase, Cochrane Library, China National Knowledge Infrastructure, Wanfang, VIP and China Biomedical Literature Database.

**Results:**

This scoping review included 15 studies and identified 18 assessment tools, falling into four categories: 8 anthropometric tools, 7 performance-based test tools, 1 questionnaire, and 2 calculation formulas. ALM/SMI is the gold standard for muscle mass; TMT enables early screening via routine CT/MRI with no extra burden. HGS is preferred for muscle strength. CC and SARC-F suit large-scale initial screening. The validated ALM prediction formula provides an alternative when instrumental measurement is unavailable.

**Conclusion:**

The results of our study can help healthcare professionals to rationally select the appropriate and optimal SRS assessment tools according to the actual situation.

## 1 Introduction

Stroke has been the second leading cause of death and the third leading cause of disability worldwide (1). With the accelerated aging of the population and lifestyle changes, the incidence, mortality, and disability of stroke are on the rise globally (2). Skeletal muscle is the main functional organ of post-stroke disability (3). Post-stroke patients experience extensive muscle mass changes such as paralysis and wasting, spasticity, impaired feeding, and intestinal absorption-related atrophy, and are therefore more susceptible to sarcopenia (4).

Sarcopenia is a skeletal muscle disorder involving the loss of skeletal muscle mass and function and is further associated with falls, functional decline, frailty, and mortality (5). Stroke-related sarcopenia (SRS), a subtype of sarcopenia secondary to stroke, is a clinical syndrome characterized by reduced skeletal muscle mass, diminished muscle strength, and impaired physical function, arising from the combined effects of neurological injury, limited mobility, metabolic dysregulation, and underlying health status post-stroke (6). Compared with age-related sarcopenia, SRS has an acute onset and rapid progression, with more prominent muscle atrophy on the hemiplegic side and a strong correlation between functional impairment and neurological deficits (6, 7). Existing studies report the prevalence of SRS ranges from 16.8 to 60.3%, and sarcopenia is strongly associated with multiple adverse clinical outcomes such as changes in cognition, depression, loss of functional capacity, decreased quality of life, falls, hospitalizations, and mortality (8). Furthermore, SRS accelerates the progression of muscle atrophy and exacerbates the patient’s dysfunction, resulting in a reduction in mobility and impeding the rehabilitation process (9). SRS not only seriously affects the quality of life of older adults, but also imposes a heavy financial burden on families and society in terms of follow-up medical care (10).

The development process of SRS is generally hidden and not easily detected by patients until the occurrence of the above-mentioned adverse consequences (11). Early assessment in stroke patients is essential to prevent the onset or progression of SRS (12). Researchers around the world have explored different perspectives on tools that can be used to assess sarcopenia (13). Current internationally accepted sarcopenia diagnostic frameworks, including the European Working Group on Sarcopenia in Older People 2 (EWGSOP2) and the 2019 consensus of the Asian Working Group for Sarcopenia (AWGS), define three evaluation domains: muscle mass, muscle strength, and physical performance. Appendicular lean mass/skeletal muscle mass index (ALM/SMI) measured via DXA or BIA serves as the gold standard for muscle mass quantification, while handgrip strength (HGS) and gait speed tests are used to assess muscle strength and physical performance, respectively (14, 15).

Stroke patients have clinical difficulties in executing several tests of muscle strength and physical performance among the criteria for sarcopenia due to paralysis, cognitive impairment, abnormal muscle tone, impaired sensation, poor balance, and proprioception deficits (16). Specifically, DXA requires bulky equipment and cannot be performed bedside, HGS measurements are compromised by hemiplegic dysfunction, and gait speed tests are unfeasible for stroke survivors with balance disorders or impaired independent ambulation. Given the limited applicability of conventional gold-standard tools among stroke patients, specialized SRS assessment tools that patients can cooperate with are urgently needed. Therefore, it is critical to identify sarcopenia assessment tools and protocols suitable for stroke populations. Recent research has focused on the development and clinical application of SRS evaluation instruments (4, 17). To our knowledge, no prior study has systematically summarized all available SRS assessment tools.

Nevertheless, the pathogenesis of post-stroke sarcopenia is multidimensional and complex. It may stem from pre-stroke frailty, and also arises from the combined effects of post-stroke immobility, inadequate nutritional intake, systemic inflammatory response, neurological deficits, and age-related degenerative changes. Accordingly, sarcopenia detected after stroke cannot be entirely attributed to stroke itself, and clear pathophysiological criteria to distinguish the two remain absent. Given such complexity, this scoping review does not aim to identify stroke-specific sarcopenia assessment tools. Instead, it addresses a more clinically actionable question: which validated or promising sarcopenia assessment tools can deliver effective and feasible evaluations specifically for stroke patients.

## 2 Materials and methods

### 2.1 Protocol and registration

This protocol was developed using the methodological framework for scoping reviews proposed by Arksey and O’Malley (18). We used the Preferred Reporting Items for Systematic Reviews and Meta-analysis: Extension for Scoping Review reporting guidelines for the full report (19). The protocol for this review was registered with the Open Science Framework: https://osf.io/2s4uj (accessed on 1 May 2026).

### 2.2 Objectives of the study

The primary review question was: What assessment tools are available for SRS? Two specific objectives were identified: (i) To identify SRS assessment tools that have been used in clinical practice. (ii) To map the characteristics of these tools, including method, instrumentation, process, criteria for positive screen, metrological characteristics, assessment field.

### 2.3 Eligibility standards

Literature inclusion criteria: (i) the study subjects were confirmed stroke patients; (ii) the study topic contain “assessment of SRS”; (iii) the assessment tool for SRS was clearly stated in the literature; (iv) the type of study was an original study, including cross-sectional, longitudinal, cohort, and case-control studies. Exclusion criteria: (i) duplicate inclusion; (ii) unavailability of full text; (iii) non-Chinese or English literature; (iv) conference abstracts.

### 2.4 Information sources and search strategy

The search strategy was based on the three-step method recommended by the JBI. In the first step, a preliminary search was conducted in PubMed and China National Knowledge Infrastructure. The search terms in the titles, abstracts, and full text of the retrieved studies were analyzed to ensure the final search terms. Next, Five English databases including PubMed, Cumulative Index to Nursing and Allied Health Literature, Web of Science, Embase, Cochrane Library, and four Chinese databases including China National Knowledge Infrastructure, Wanfang, VIP and China Biomedical Literature Database were systematically searched from inception to 28 June 2026. No date limits were applied in the search in an attempt to capture as many relevant studies as possible. Finally, reference lists were checked in all identified articles for additional studies.

The same search strategy and search words were conducted in all databases, four different combinations were used: (1) stroke, (2) sarcopenia, (3) assessment tools, and (4) 1 and 2 and 3. The specific search terms are shown in Table 1, and the complete PubMed search strategy is presented in Table 2.

**TABLE 1:** Concept groups and search terms.

Concept groups	Search terms
Stroke	Stroke*/cerebral apoplexy/cerebrovascular accident/cerebrovascular disease/ cerebral embolism/cerebral ischemia*/cerebral hemorrhage*/cerebral infarction*/ cerebrovascular disorder */cerebral vascular event/transient ischaemic attack/post stroke/after stroke
Sarcopenia	Sarcopenia/sarcopenic/muscle loss/muscle weakness/muscular atrophy
Assessment tools	Tool*/scale*/instrument*/index*/questionnaire*

*A truncation symbol retrieving all suffix variants of the root word in PubMed search strategy.

**TABLE 2:** The search query for PubMed database.

Search	Query
**Pubmed**	
#1	((“Stroke”[Mesh]) OR (Stroke*[Title/Abstract] OR “cerebral apoplexy”[Title/Abstract] OR “cerebrovascular accident”[Title/Abstract] OR “cerebrovascular disease”[Title/Abstract] OR “cerebral embolism”[Title/Abstract] OR “cerebral ischemia*”[Title/Abstract] OR “cerebral hemorrhage*”[Title/Abstract] OR “cerebral infarction*”[Title/Abstract] OR “cerebrovascular disorder *”[Title/Abstract] OR “cerebral vascular event”[Title/Abstract] OR “transient ischaemic attack”[Title/Abstract] OR “post stroke”[Title/Abstract] OR “after stroke”[Title/Abstract]))
#2	(“Sarcopenia”[Mesh]) OR (sarcopenia [Title/Abstract] OR sarcopenic [Title/Abstract] OR “muscle loss”[Title/Abstract] OR “muscle weakness”[Title/Abstract] OR “muscular atrophy”[Title/Abstract])
#3	(Tool*[Title/Abstract] OR scale*[Title/Abstract] OR instrument*[Title/Abstract] OR index*[Title/Abstract] OR questionnaire*[Title/Abstract])
	#1 and #2 and #3

*A truncation symbol retrieving all suffix variants of the root word in PubMed search strategy.

Grey literature including conference abstracts, dissertations and unpublished research reports was excluded from this review for three reasons. First, this study primarily intends to systematically summarize clinically validated SRS assessment tools, and peer-reviewed published papers provide more reliable research quality and evidence. Second, grey literature generally lacks complete methodological descriptions and psychometric data of assessment tools, failing to support the extraction of measurement characteristics required in this review. Third, grey literature on SRS is scattered, rendering comprehensive retrieval impractical and likely introducing greater selection bias.

### 2.5 Selection of the sources of evidence

Studies retrieved from the database were exported to EndNote X9, which facilitated data management, removal of duplicates, and independent screening by evaluators. Two team members independently screened the titles and abstracts of retrieved results for eligibility and resolved conflicts with a third reviewer. Only studies that were relevant to the research topic proceeded to the next stage of full-text screening. If the relevance of the study was not clear from the abstract, the entire article was reviewed.

### 2.6 Data extraction

Excel was used by two researchers separately to extract data from eligible studies to ensure accuracy and extracted the following data: authors, year of publication, country and language, study design, sample size, research population (range of age), research tools, reference tools, setting, and assessment time. For eligible articles with missing key data or ambiguous descriptions, the research team will contact the corresponding authors via email to verify and supplement relevant information. If no response is received within 14 working days after email delivery, only publicly available data reported in the published full texts will be adopted.

### 2.7 Quality assessment

The quality of the studies included in this review was not accessed because there are no mandatory requirements for scoping reviews.

## 3 Results

**FIGURE 1:**
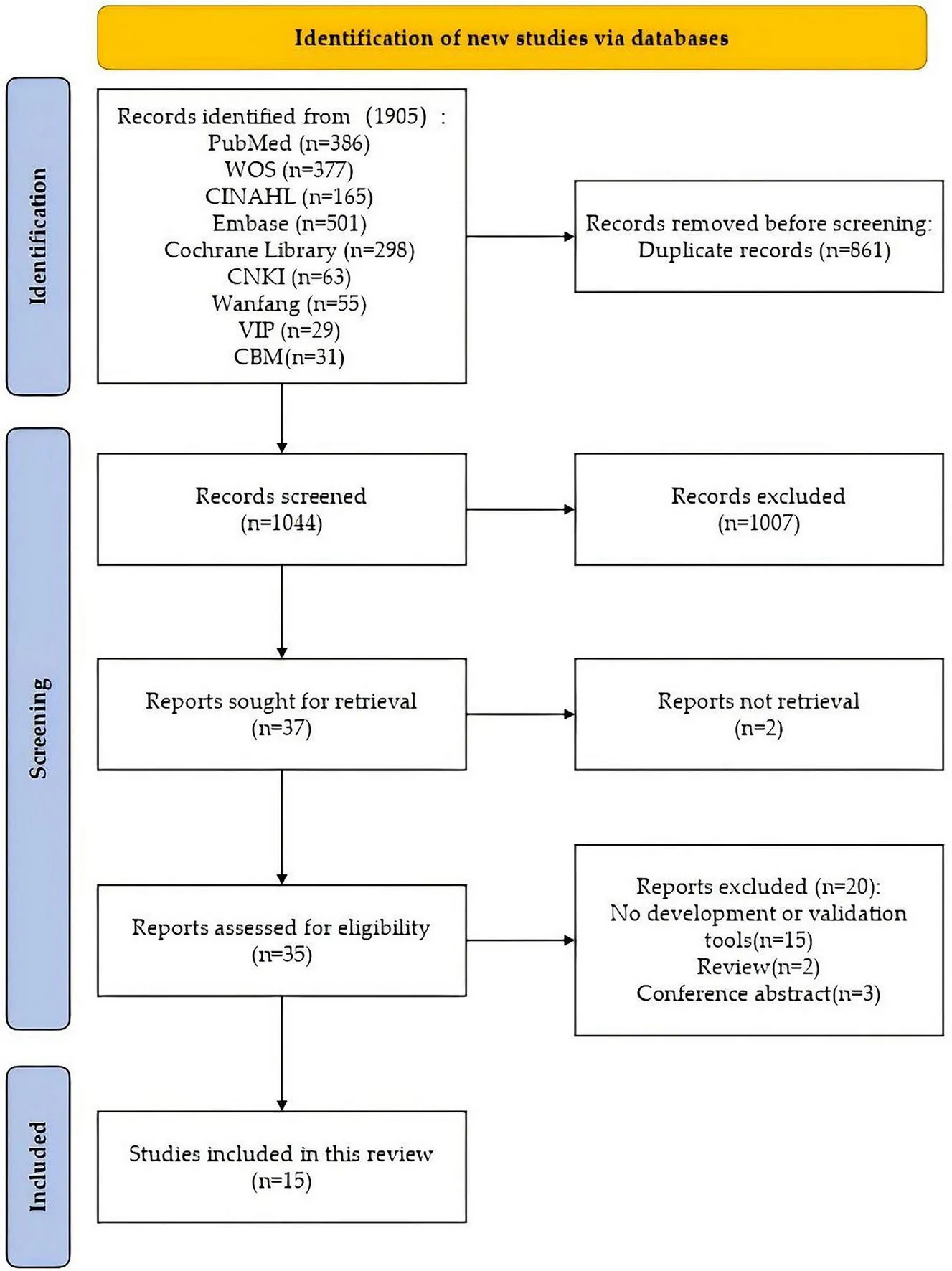
Flowchart of the study selection process.

A total of 1,905 articles were identified through electronic database searching (PubMed: 386, Web of Science: 377, Cumulative Index to Nursing and Allied Health Literature: 165, Embase: 501, Cochrane Library: 298, China National Knowledge Infrastructure: 63, Wanfang: 55, VIP: 29, China Biomedical Literature Database: 31. Following the removal of 861 duplicates, 1044 records were screened for inclusion. From these, 1007 articles were excluded after reading the title and abstract, which left 37 articles to be assessed for eligibility. 22 articles were excluded after reading the full text for the following reasons: the purpose of the study was not to develop or apply SRS assessment tools (*n* = 15), review (*n* = 2), conference abstract (*n* = 3), and unable to access the full text (*n* = 2). Finally, 15 articles were included in the review. The flow diagram of the study selection process is shown in Figure 1.

### 3.1 Characteristics of included studies

Table 3 reveals the characteristics of eligible articles in this scoping review. Thirteen papers were included that were published in 2020–2026. These studies were from 4 different countries, including Japan (*n* = 7) (20–26), South Korea (*n* = 5) (27–31), China (*n* = 2) (32, 33), and Spain (*n* = 1) (34). Twelve articles were in English, and one was in Chinese. The majority of studies were cross-sectional (*n* = 9) (20, 22, 24, 26, 27, 29, 30, 32), and the rest were retrospective cohort studies (*n* = 5) (21, 23, 25, 28) and prospective cohort studies (*n* = 1) (31). Fifteen studies involved 4,103 subjects, and the majority of studies recruited more than 100 subjects (*n* = 11) (20–25, 29–31). Two studies focused on acute stroke patients (21, 28) and one study focused on chronic stroke patients (32). The age of the research population ranged from 25–96 years, and 3 studies focused on elderly stroke patients over 65 years (20, 22, 24). Each study referred to 1–6 assessment tools, 10 studies used a single assessment tool for SRS (20, 22–26, 28, 29, 31), and 5 studies used a combination of several assessment tools (21, 27, 30, 32). In addition, the majority of studies reported assessment tools as well as reference tools as criteria (*n* = 11) (20–24, 26–30), and a few studies did not report reference tools (*n* = 4) (25, 31, 32). The settings in which these assessments were performed were mostly in clinical hospitals (*n* = 14) (20–31), particularly rehabilitation units or rehabilitation hospitals (*n* = 8) (23, 24, 26–30), and to a lesser extent in the community (*n* = 2) (32). All studies reported the time point of assessment.

**TABLE 3:** The characteristics of eligible articles in this scoping review.

Authors (Reference)	Country (Language)	Study design	Samplesize	Research population (Range of age)	Research tools	Reference tools	Setting	Assessment time
Nozoe et al. (20)	Japan (English)	Cross-sectional study	289	Elderly stroke patients (69–83)	TMT	SARC-F	Neurosurgical Hospital	Stroke attack ≤ 7 days
Abe et al. (21)	Japan (English)	Retrospective cohort study	308	Acute stroke patients (60–86)	CC HGS	SMI HGS	Acute care hospital	Stroke attack ≤ 7 days
Arnal-Gómez et al. (34)	Spain (English)	Cross-sectional study	34/34	Chronic stroke patients/ non-stroke patients (51–71)	SARC-F CC HGS 5STS SPPB 10MWT	–	Community	Stroke attack ≥ 6 months
Sato et al. (22)	Japan (English)	Cross-sectional study	211	Stroke patients (65–83)	Phase Angle	SMI HGS	Acute care hospital	Stroke attack ≤ 7 days
Choi et al. (27)	Korea (English)	Cross-sectional study	30	First-time stroke patients (53–80)	TRF	ALM	Department of Rehabilitation Medicine	Admission ≤ 7 days
Jung et al. (28)	Korea (English)	Cross-sectional study	40	First-time stroke patients (51–83)	TA MRC BBS	ALM HGS 4MGS	Department of Rehabilitation Medicine	Admission
Yao et al. (32)	China (English)	Cross-sectional study	364	Stroke patients (48–69)	CC SARC-F Ishii’s score	SMI HGS	Department of Rehabilitation Medicine	Stroke attack 2 weeks to 6 months
Nishioka et al. (23)	Japan (English)	Retrospective cohort study	628	Stroke patients (60–86)	CC	SMI HGS	rehabilitation wards of a rural private hospital	Stroke attack ≤ 60 days
Park et al. (29)	Korea (English)	Retrospective cohort study	25	Acute stroke patient with hemiparesis (57–82)	QMT	SMI	Department of Rehabilitation Medicine	Admission ≤ 7 days
Nagano et al. (24)	Japan (English)	Cross-sectional study	465	Elderly stroke patients (70–85)	TMT	SMI HGS	Rehabilitation Hospital	Admission
Katsuki et al. (25)	Japan (English)	Retrospective cohort study	298	Stroke patients (25–96)	TMT TMA	–	Hospital	Admission
Han et al. (30)	Korea (English)	Cross-sectional study	244	Stroke patients (50–84)	TMT TMA	SMI	Department of Rehabilitation Medicine	Admission
Li et al. (33)	China (Chinese)	Prospective cohort studies	265	Stroke patients (28∼92)	TMT	–	Hospital	Admission
Kang et al. (31)	Korea (English)	Cross-sectional study	120	Stroke patients ( ≥ 50)	HGS 5STS SMI SPPB BBS 10MWT	–	Rehabilitation centers, hospitals and communities	Stroke onset of at least 6 months
Hori et al. (26)	Japan (English)	Retrospective cohort study	748	Stroke patients (61∼85)	A validated prediction equation (ALM = 0.485 × 0.998^∧^ age × 0.814^∧^ [female] × 1.006^∧^height × weight^∧^0.680)	BIA-derived skeletal muscle mass	Rehabilitation hospitals	At hospital discharge

TMT, temporal muscle thickness; SARC-F, the SARC-F questionnaire; CC, calf circumference; HGS, handgrip strength; SMI, skeletal mass index; 5STS, five-times sit-to-stand test; SPPB, the short physical performance battery; 10MWT, the 10 m walk test; TRF, the thickness of the rectus femoris; ALM, appendicular lean mass; TA, tibialis anterior; MRC, the medical research council sum score; BBS, Berg Balance Scale; 4MGS, 4-meter gait speed; QMI, quadriceps muscle thickness; TMA, temporal muscle area; -, Information not reported.

### 3.2 Assessment tools of SRS

This review extracted 18 SRS assessment tools from 15 papers, including appendicular lean mass (ALM)/skeletal mass index (SMI) (*n* = 10), handgrip strength (HGS) (*n* = 8), temporal muscle thickness (TMT) (*n* = 5), calf circumference (CC) (*n* = 4), the SARC-F scores (*n* = 3), temporal muscle area (TMA) (*n* = 2), the thickness of the rectus femoris (TRF) (*n* = 1), tibialis anterior (TA) (*n* = 1), quadriceps muscle thickness (QMI) (*n* = 1), Phase Angle (*n* = 1), the medical research council sum score (MRC) (*n* = 1), five-times sit-to-stand test (5STS) (*n* = 2), the short physical performance battery (SPPB) (*n* = 2), 4-meter gait speed (4MGS) (*n* = 1), the 10 m walk test (10MWT) (*n* = 2), Berg Balance Scale (BBS) (*n* = 2) and Ishii’s score (*n* = 1). Depending on the type of assessment, it can be categorized into four instrument types: anthropometric tools (*n* = 8), activity test tools (*n* = 7), questionnaire (*n* = 1), and formulaic calculation (*n* = 2). Table 4 shows the Characteristics of the assessment tools.

**TABLE 4:** The characteristics of the assessment tools.

Types	Tools	Instrument actions	Process	Criteria for positive screen	Metro logical characteristics	Assessment field	Number of use
Anthropometric tools							
	ALM/ SMI	DXA/BIA	ALM: Direct measurement; SMI: ALM/height^2^ (kg/m^2^)	⋅ALM: Men < 19.75 kg; Women < 15.02 kg; ⋅SMI: Men < 7.0 kg/m^2^; Women < 5.7 kg/m^2^	–	Muscle mass	10
	TMT	CT	Manual measurement based on CT results. The TMT values for the left and right sides are added and divided by 2 to get the average TMT for each patient	All < 4.625 mm Men < 3.83 mm and Women < 2.78 mm	Sensitivity:0.54, Specificity:0.75 ⋅Men: Sensitivity:0.642 Specificity:0.750 ⋅Women: Sensitivity: 0.660 Specificity: 0.567	Muscle mass	5
	CC	Tape measure	Sitting or lying, bend your knees 90 degrees and use a soft ruler to measure the thickest part of both legs. Take the maximum value.	Men < 31–33 cm; Women < 30–32 cm.	⋅Men: Sensitivity:0.789–0.815 Specificity:0.707–0.957 ⋅Women: Sensitivity:0.839–0.847 Specificity:0.630–0.818	Muscle mass screening	4
	TMA	CT	Manual measurement based on CT results.	< 200 mm	–	Muscle mass	2
	TRF	Ultrasound	Cross-sectional ultrasound images were recorded on half of the distance from the anterior superior iliac spine to the apex of the patella.	–	The EI to TRF ratio in the paretic side in the sarcopenia group was significant	Muscle mass	1
	TA	Ultrasound	TA was assessed using an electronic caliper at a quarter of the distance from the lower patella to the lateral ankle bone.	Men ≤ 2.790 cm Women ≤ 2.055 cm	⋅men: Sensitivity:1.000 Specificity:0.667 ⋅Women: Sensitivity:1.000 Specificity:0.937	Muscle mass	1
	QMI	Ultrasound	The thickness of the medial muscle at the anterior superior iliac spine and the proximal patella was measured using an electronic caliper.	–	The QMT and ALM index showed a positive correlation	Muscle mass	1
	Phase Angle	BIA	Phase angle = arctangent (Xc/ R) £ (180/p), where R is the resistance of the right half of the body and Xc is the reactance measured at 50 kHz.	Men ≤ 5.28 Women ≤ 4.62	⋅Men: Sensitivity:0.900 Specificity:0.827 ⋅Women: Sensitivity:0.914 Specificity:0.694	Muscle mass	1
Activity test tools							
	HGS	Grip dynamometer	Alternately measure the maximum grip strength three times on each arm, recording the highest measurement.	Men < 27 Women < 16	ICC ≥ 0.86–0.95	Muscle strength	8
	MRC	–	The muscle strength scores of bilateral shoulder abductors, elbow flexors, wrist extensors, hip flexors, knee extensors and ankle dorsiflexors were evaluated. Each score is 0–5 points, the total score is 0–60 points.	Men ≤ 54.00 Women ≤ 53.42	⋅Men: Sensitivity:0.778 Specificity:0.500 ⋅Women: Sensitivity:0.801 Specificity:0.639	Muscle strength	1
	5STS	Stopwatch	The time (in seconds) taken to complete 5 repetitions of sitting and standing in a standard-height chair.	≥ 12 s	–	Muscle strength	2
	SPPB	Stopwatch	Three parts are involved: balance, gait and leg strength. Each section is scored from 0 to 4, with a total score of 12.	≤ 8	–	Physical performance	2
	4MGS	Stopwatch	Walk at a speed of 4 meters on a flat road.	< 1.0 m/s	–	Physical performance	1
	10MWT	Stopwatch	Walk 10 meters and take the walking speed at 6 m in the center.	< 0.8 m/s	ICC: 0.94	Physical performance	2
	BBS	–	There are 14 sections that assess the balance function. Each section is 0–4 points, and the total score is 56 points.	≤ 41	Sensitivity:0.781 Specificity:1.000	Physical performance	2
Questionnaire							
	SARC-F	–	Five items are involved: strength, help walking, getting up from a chair, climbing stairs, and falling. Each entry has a score of 0–2, with a total score of 10.	≥ 5	Sensitivity:0.947 Specificity:0.400	Physical performance	3
Formulaic calculation							
	Ishii’s score	tape measure	Men = 0.62 × (age-64)-3.09 × (HGS-50)-4.64 × (CC-42); Women = 0.80 × (age-64)-5.09 × (HGS -34)-3.28 × (CC-42).	≤ 118	Sensitivity:0.901 Specificity:0.360	Physical performance	1
	ALM	–	ALM = 0.485 × 0.998^∧^age × 0.814^∧^[female] × 1.006^∧^height × weight^∧^0.680	SMI: Men < 7.0 kg/m^2^; Women < 5.7 kg/m^2^	⋅Men: Sensitivity: 0.830 Specificity: 0.650 ⋅Women: Sensitivity:0.960 Specificity:0.550	Muscle mass screening	1

ALM, appendicular lean mass; SMI, skeletal mass index; DXA, Dual-energy X-ray Absorptiometry; BIA, Bioelectrical Impedance Analysis; CC, calf circumference; TMT, temporal muscle thickness; CT, Computed Tomography; TMA, temporal muscle area; TRF, the thickness of the rectus femoris; TA, tibialis anterior; QMI, quadriceps muscle thickness; HGS, handgrip strength; MRC, the medical research council sum score; 5STS, five-times sit-to-stand test; SPPB, the short physical performance battery; 4MGS, 4-meter gait speed; 10MWT, the 10 m walk test; BBS, Berg Balance Scale; SARC-F, the SARC-F questionnaire; –, Information not reported.

#### 3.2.1 Anthropometric tools

Assessment tools for anthropometric types included AML/SMI, CC, TMT, TMA, TRF, TA, QMI and Phase Angle. 10 studies used ALM/SMI to assess muscle mass in SRS (21–24, 26–30). ALM was the sum of lean mass from both arms and legs, Rely on Dual-energy X-ray Absorptiometry (DXA) or Bioelectrical Impedance Analysis (BIA) to measure. The SMI was obtained by adjusting ALM with height. It has been suggested that SMI is superior to CC in assessing SRS in patients with acute stroke21. Four studies have used CC as an assessment tool for SRS (21, 23, 30, 32). CC does not require instrumentation to perform the measurements and is rapid, noninvasive, and inexpensive (21). One study determined the agreement between CC and low SMI measured by bioelectrical impedance analysis (23). A Chinese study showed that CC had higher specificity than SARC-F and Ishii’s scores in screening for SRS (30), 3 studies used TMT and/or TMA to assess muscle mass (27, 29, 31). Stroke patients routinely require testing Computed Tomography (CT) or Magnetic Resonance Imaging (MRI) for definitive diagnosis, and TMT and TMA can be measured using CT or MRI taken on admission without the need for additional tests and higher costs. The criteria for TMT have good sensitivity and specificity in assessing SRS in elderly acute stroke patients (20, 24). Ultrasound has also been validated as an assessment tool for SRS. Ultrasound has the advantage of being low-cost, radiation-free, convenient, and available at the bedside, and is less costly and more readily available than CT and MRI. Ultrasound-measured TRF is a key indicator of SRS in the early stages of subacute stroke. In assessing muscle mass, ultrasound-measured TA muscle thickness and quadriceps muscle thickness have a high correlation with ALM/SMI (27). One study redefined the threshold of phase angle for the assessment of SRS (22), and showed that phase angle is a useful indicator for identifying skeletal muscle loss in acute stroke patients, and was significantly correlated with HGS, SMI, and CC in elderly acute stroke patients.

#### 3.2.2 Activity testing tools

A total of 7 activity testing instruments were involved in this study, including HGS, 5STS, MRC, SPPB, BBS,4MGS, and 10MWT. Among them, HGS, 5STS, and MRC scores were used to assess muscle strength, and SPPB, BBS,4MGS, and 10MWT were used to assess physical performance. 8 studies used the HGS to assess muscle strength (21–24, 27, 30, 32), 1 study showed a difference in 5STS scores between chronic stroke and non-stroke patients (32), and 1 study used the MRC total score test instead of the HGS to measure muscle strength (27). Only 2 studies mentioned tools for assessing physical performance, with 1 study examining whether the SPPB and 10MWT could be used to assess physical performance in patients with chronic stroke (32), and the other study suggested that the BBS could be used in place of the 4MGS for the assessment of somatic function (27).

#### 3.2.3 Questionnaire

There was 1 questionnaire-type assessment tool in this study, the SARC-F scale. 3 studies used the SARC-F scale to assess SRS (20, 30, 32). Of these, 1 study redefined new threshold values for the use of the SARC-F scale for clinical screening for SRS (30), and 1 study validated the SARC-F scale for use in a community setting for SRS (32).

#### 3.2.4 Formula calculation

Assessment tool that was calculated using a formula in this study was the Ishii’s score. 1 study compared the validity of the Ishii’s score, CC and SARC-F scales in screening for SRS and redefined the threshold value of the Ishii’s score for judging SRS (30).

## 4 Discussion

This study aimed to clarify what validated tools for the assessment of SRS were available after a comprehensive scoping review. Among 13 studies included in this Scoping Review, 17 different dietary assessment tools were used: ALM/SMI, HGS, TMT, CC, the SARC-F scores, TMA, TRF, TA, QMI, Phase Angle, MRC, 5STS, SPPB, 4MGS, 10MWT and BBS. Depending on the type of assessment, it can be categorized into four instrument types: anthropometric tools (*n* = 8), activity test tools (*n* = 7), questionnaire (*n* = 1), and formulaic calculation (*n* = 1).

Most of the studies included in this review were conducted in Asia (*n* = 14), especially Japan (*n* = 7). In Asia, Japan has the highest incidence of stroke and the lowest mortality rate, and the large number of stroke survivors has led researchers to focus on the assessment and identification of SRS as a stroke sequela, which may partially justify studies involving more SRS assessment tools (33). The rapid development of rehabilitation medicine in Japan has established a unique system of convalescent rehabilitation wards (CRW) to identify SRS early and provide timely rehabilitation interventions, which may be helpful to the research of SRS and sarcopenia to some extent (34).

Both the Asian Working Group for Sarcopenia (AWGS) and the European Working Group on Sarcopenia in Older People (EWGSOP) specify that sarcopenia assessment comprises three core domains: muscle mass, muscle strength and physical performance (35). Most existing tools for stroke-related sarcopenia (SRS) are adapted from these general assessment frameworks, with no fully independent SRS-specific instruments developed to date. While they share identical core assessment dimensions, distinctions lie in clinical adaptability. General sarcopenia tools are designed for older adults with preserved physical function and demand high participant cooperation. In contrast, SRS assessments accommodate stroke patients’ clinical manifestations including hemiplegia, cognitive impairment and mobility limitation, thus prioritizing objective instrumental measurements and simple physical examinations with population-specific adjustments to diagnostic cutoffs and testing procedures. Representative examples include measuring temporal muscle thickness (TMT) via routine CT scans for bedridden patients, and adopting the MRC scale instead of handgrip strength to evaluate muscle strength in hemiplegic individuals (36). A global stroke screening data for China shows that about 40% of patients will leave moderate dysfunction and 15%–30% of patients with severe disability (37). Physical impairments in stroke patients may impair their capacity to fully engage in all forms of testing, particularly those involving physical activities. In contrast to age-related sarcopenia, research on SRS assessment tools emphasizes the quantification of muscle mass (38). This approach boasts a distinctive advantage: it necessitates minimal patient cooperation, as it primarily relies on instrumental measurements (17). The majority of studies within this scoping review have solely evaluated muscle mass and muscle strength. However, stroke patients who are in recovery or capable of completing physical activity assessments are still encouraged to receive a more comprehensive, multi-layered assessment that includes physical activity (39).

Despite the existence of multiple assessment tools for stroke-associated sarcopenia, each method possesses its specific advantages, limitations and applicable conditions. The results of the study yielded 17 SRS assessment tools, which can be divided into four categories depending on the assessment method.

The most commonly used are anthropometric tools, with a total of 8 anthropometric tools mentioned. Since anthropometric tests do not require the subject to cooperate in cognitive and physical activities, these types of tools are friendly to stroke patients who experience mobility impairments, cognitive deficits, and hemiparesis (40). However, most anthropometric tools require instrumentation to perform the measurements, so the availability and economic cost of these measurement devices need to be considered for practical applications. ALM/SMI is the most mentioned anthropometric tool, it is AWGS, EWGSOP and the Foundation for the National Institutes of Health (FNIH) Biomarkers Consortium recognized as the gold standard for measuring muscle mass and is already widely used in the clinic (35, 36, 41). DXA and BIA, as the primary techniques for measuring ALM, each possess their strengths and weaknesses. DXA offers precise quantification of muscle mass but is associated with higher costs and more complex operation procedures (42). In contrast, BIA is more convenient; however, its accuracy may be influenced by various factors, such as water content and body fat distribution (43). Future research should further explore ways to optimize the accuracy of BIA across different populations, aiming to reduce assessment costs. TMT is a human testing tool mentioned only second to ALM/SMI, which can measure muscle mass through computed tomography (CT), magnetic resonance imaging (MRI) and ultrasound, and has attracted extensive attention from scholars at home and abroad in recent year (20). CT is a routine examination for stroke patients, so the assessment of SRS based on TMT will not bring additional examination pressure and financial burden to patients, and can be used for the early screening of SRS (44). In addition, CC measurement is also extensively utilized in various settings due to its instrument-free requirement, rapid execution, non-invasive nature, and cost-effectiveness (45). These attributes render CC particularly advantageous for applications in community health programs, nursing homes, and large-scale population screenings for stroke patients. However, it is noteworthy that CC measurements can be influenced by edema, necessitating careful consideration to mitigate the potential interference of edema in the assessment process (46).

The most commonly used activity testing tool is the HGS, which is widely used to measure muscle mass because of its simplicity and ease of measurement by simply using a HGS meter to obtain results. Although HGS is reliable and responsive and correlates with functional status and motor performance in stroke patients, this test does not correlate well with overall muscle strength because it uses only relatively small muscle groups (47). There have also been some studies suggesting that the HGS on the mildly hemiplegic side may reveal a floor effect (35, 48). The MRC score can be used as an alternative assessment tool when the subject is unable to complete the HGS. The MRC score is also a reliable assessment tool used to measure muscle strength in a variety of clinical situations, including stroke, and covers a larger group of muscles than the HGS and does not require equipment such as ergometers (49).

The SARC-F scale was the only questionnaire-type instrument used in this study, and it was also a common screening tool for age-related sarcopenia. The AWGS recommends that a SARC-F score ≥ 4 identify possible sarcopenia and that further screening be performed. However, some studies have suggested that a SARC-F score ≥ 5 may identify the presence of SRS (30). The new threshold is currently less well-studied, and more studies could be conducted to validate the threshold in the future (50).

Overall, three core commonalities exist in the application of current SRS assessment tools. First, ALM/SMI is widely adopted as the reference gold standard for muscle mass evaluation to verify the diagnostic validity of various simplified tools, establishing its benchmark role in SRS assessment. Second, constrained by the general functional limitations of stroke survivors, most studies prioritize anthropometric tools requiring minimal active patient cooperation, focusing assessments on muscle mass and strength, while physical performance tools are less frequently utilized. Third, studies conducted in Asia generally favor non-invasive, low-cost, bedside-accessible measurement methods that fit well within routine stroke clinical workflows.

Marked heterogeneity in tool selection is observed across studies. Stratified by stroke phase, objective metrics such as TMT, phase angle and CC are predominantly used in acute-phase research to accommodate bedridden patients with severe dysfunction. By contrast, chronic and community-based studies more frequently incorporate muscle strength and functional assessments including HGS, 5STS, SPPB and SARC-F, enabling multidimensional evaluation. Furthermore, studies vary greatly in assessment comprehensiveness: most only implement single-domain measurements, while merely a small number of chronic-stroke studies cover all three domains of muscle mass, muscle strength and physical performance. To date, no unified consensus has been reached on a standardized assessment framework for SRS.

## 5 Conclusion

According to our review, there are 17 validated assessment tools for SRS. Different assessment tools have different methods and scopes of application, and it is recommended that healthcare professionals choose the most appropriate assessment tool according to the actual situation to obtain objective assessment results. Meanwhile, the assessment of SRS has not yet been popularized in clinical settings and needs to be further promoted in clinical and community settings in the future.

## 6 Limitations

There are some limitations to this review. First, although Chinese and English are the dominant publication languages in biomedicine covering most high-quality global evidence, relevant studies published in other languages are inevitably omitted. Second, systematic searches across nine databases yielded only 15 eligible studies, most of which were conducted in Japan and South Korea. This may limit the generalizability of our findings to populations and cultural settings worldwide. Additionally, several included SRS assessment tools remain in the early stages of development or validation, lacking large-sample, multicenter clinical trials to confirm their efficacy.

## 7 Prospective

Future research should focus on further validating and optimizing assessment tools for stroke-related sarcopenia, particularly by testing their reliability and validity in multicenter, large-sample clinical settings and establishing more universal diagnostic cutoffs. Furthermore, efforts should be made to explore how these assessment tools can be effectively disseminated in early rehabilitation and community settings to improve screening coverage and timely intervention. Technically, further utilization of objective measures such as imaging and bioelectrical impedance can reduce reliance on patient compliance, with particular emphasis on developing low-burden assessment methods suitable for patients with paralysis or cognitive impairment. Furthermore, cross-cultural and cross-regional collaborative research will help validate the applicability of current tools in diverse populations and promote international consensus. The ultimate goal is to improve muscle health management and enhance long-term quality of life and functional outcomes in stroke patients through early, accurate, and efficient assessment.

Furthermore, future research could focus on integrating multiple assessment tools to construct a multidimensional, staged assessment system to more comprehensively capture the trajectory of changes in muscle mass, strength, and physical function after stroke, providing a more robust basis for clinical decision-making.

## Data Availability

The original contributions presented in this study are included in the article, further inquiries can be directed to the corresponding author.
